# 快速HE染色在周围型肺癌穿刺活检快速现场细胞学评价中的应用

**DOI:** 10.3779/j.issn.1009-3419.2023.101.24

**Published:** 2023-08-20

**Authors:** Jian HE, Guilan XIA, Shiping WANG, Kun CHEN

**Affiliations:** ^1^650300 昆明，安宁市第一人民医院呼吸与危重症医学科; ^1^Department of Pulmonary and Critical Care Medicine, Anning First People's Hospital, Kunming 650300, China; ^2^650300 昆明，安宁市第一人民医院病理科; ^2^Department of Pathology, Anning First People's Hospital, Kunming 650300, China; ^3^650300 昆明，安宁市第一人民医院医学影像中心; ^3^Imaging Medical Center, Anning First People's Hospital, Kunming 650300, China; ^4^200040 上海，复旦大学附属华山医院宝山院区检验医学科; ^4^Department of Laboratory Medicine, Baoshan District of Huashan Hospital, Fudan University, Shanghai 200040, China

**Keywords:** 肺肿瘤, 周围型肺癌, CT引导肺活检, 快速现场细胞学评价, 快速HE染色, Lung neoplasms, Peripheral lung cancer, CT-guided lung biopsy, Cytological rapid on-site evaluation, Rapid HE staining

## Abstract

**背景与目的:**

快速现场评价（rapid on-site evaluation, ROSE）是在活检术中同步实施的标本快速细胞学染色和评价技术。迪夫（Diff-Quik, DQ）染色是快速现场细胞学评价（cytological ROSE, C-ROSE）最常用的染色方法，但国内大部分病理医生不使用DQ染色进行现场细胞学判读，因此难以开展C-ROSE。本研究拟在周围型肺癌穿刺活检过程中分别使用快速苏木素-伊红（hematoxylin-eosin, HE）染色和DQ染色进行C-ROSE，评估快速HE染色在C-ROSE中的应用价值。

**方法:**

对300例术前拟诊周围型肺癌患者实施胸部计算机断层扫描（computed tomography, CT）引导肺活检，随机分成两组且分别使用快速HE染色和DQ染色，进行C-ROSE判读，并对两种染色方法进行比较和评价。

**结果:**

C-ROSE与组织病理诊断符合率为96.7%。快速HE染色的中位时间为160 s，而DQ染色为120 s，两组存在统计学差异（P<0.001）。但两组的肺活检时间、组织病理符合率、细胞学标本脱片率和严重不良反应发生率均无统计学差异（P>0.05）。

**结论:**

在周围型肺癌活检过程中，两种染色方法均能满足C-ROSE要求，快速HE染色可应用于C-ROSE，且更符合国内临床需求，具有潜在的推广价值。

## Abstract (en)

**Background and objective:**

Rapid on-site evaluation (ROSE) is a technique used for simultaneous evaluation of biopsy specimens through rapid cytology staining. Diff-Quik (DQ) staining is the most commonly employed method for cytological rapid on-site evaluation (C-ROSE). However, the utilization of DQ staining for on-site cytological interpretation remains uncommon among pathologists in China, posing challenges to the implementation of C-ROSE. This study aims to assess the application of rapid hematoxylin-eosin (HE) staining and DQ staining for C-ROSE during percutaneous needle biopsy of peripheral lung cancer and evaluate the value of rapid HE staining in C-ROSE.

**Methods:**

Computed tomography (CT)-guided lung biopsies were conducted on 300 patients diagnosed with peripheral lung cancer. The patients were randomly assigned to two groups for C-ROSE using either rapid HE staining or DQ staining, and subsequently the two methods were compared and evaluated.

**Results:**

The concordance rate between C-ROSE and histopathological diagnosis was 96.7%. The median staining time for rapid HE staining was 160 s, while that for DQ staining was 120 s, representing a significant difference between the two groups (P<0.001). However, there were no significant differences observed in terms of total biopsy time, concordance rate with histopathology, cytology specimen peeling rate, and incidence of serious adverse reactions between the two groups (P>0.05).

**Conclusion:**

Both staining methods comply with C-ROSE criteria in the biopsy setting of peripheral lung cancer. Rapid HE staining is more aligned with domestic clinical requirements and holds potential for further promotion and adoption in C-ROSE.

肺癌的诊疗已经进入到个体化精准诊疗时代，活检取材不仅需满足组织形态学诊断，还要满足免疫组化、驱动基因和免疫检查点等检测需要^[1⇓-3]^，因此对临床微创取材提出了更高的要求。计算机断层扫描（computed tomography, CT）引导肺穿刺活检是诊断周围型肺癌的重要取材技术^[4,5]^，取材质量能否满足组织病理和分子诊断的需求、避免二次活检，已成为急需解决的临床问题。实时伴随活检过程的快速现场细胞学评价技术（cytological rapid on-site evaluation, C-ROSE）应运而生^[6]^。目前国际上ROSE染色技术首选推荐迪夫（Diff-Quik, DQ）染色。国内ROSE技术尚处于起步和探索阶段，绝大多数病理科医生不习惯应用DQ染色进行细胞学诊断，难以配合临床开展ROSE技术。鉴于快速苏木素-伊红（hematoxylin-eosin, HE）染色已广泛应用于术中冰冻组织的快速诊断，本研究针对周围型肺癌穿刺活检标本分别使用快速HE染色和DQ染色进行C-ROSE，并与最终病理检查结果对比，评估快速HE染色在C-ROSE中的应用价值。

## 1 资料与方法

### 1.1 一般资料

本研究纳入2021年6月至2022年12月我院收治的300例肺外周结节/阴影患者。纳入标准：（1）术前拟诊为周围型肺癌；（2）实性成分直径≥1.5 cm。入组采用信封随机分组，按照1:1分配HE染色组和DQ染色组，每组150例。入选患者均实施CT引导肺穿刺活检。本研究获得安宁市第一人民医院医学伦理委员会批准；患者术前均签署知情同意书。

### 1.2 操作方法

（1）CT引导肺穿刺活检：选择穿刺体位，在体表部位贴定位标，经过CT扫描定位和标记，并测量进针深度和进针角度。皮肤消毒和局麻，将同轴引导鞘植入病灶，拔出针芯后植入全槽活检枪穿刺切割病灶。切割活检≥3次。以快速染色结果配合CT实时指导调整穿刺活检部位、路径角度，控制活检次数。（2）快速染色：将切割组织在商品化防脱玻片进行滚片，按照分组分别实施快速HE染色或DQ染色；至少实施2次活检标本的滚片；快速HE染色方法：将涂片浸入无水甲醇中浸泡10 s，浸入苏木素染色液中60 s，水洗10 s，将涂片浸入1%碳酸锂溶液中蓝化10 s，浸入水溶性伊红染液10 s，依次80%乙醇、95%乙醇、100%乙醇各10 s，用家用电吹风吹干后，中性树脂封片后镜检。DQ染色方法：涂片在甲醇中固定10 s，轻轻甩干玻片后，依次放在DQ-A液中浸染15 s、缓冲液漂洗、DQ-B液中浸染30 s、缓冲液漂洗、轻轻甩干玻片，显微镜下判读。（3）现场细胞学判读：细胞病理学医师现场或远程判读。判读标准：见恶性肿瘤细胞判断为阳性，细胞学诊断初筛分型：非小细胞肺癌（non-small cell lung cancer, NSCLC）和小细胞肺癌（small cell lung cancer, SCLC），但不作为临床最终诊断。单张玻片恶性肿瘤细胞数量≥200个判断为取材成功。

### 1.3 评价指标

包括：与最终组织病理诊断符合率、染色和判读时间、肺穿刺活检时间、细胞学脱片（染色后细胞脱落导致无法诊断）率、严重不良反应发生率（需要置管引流的气胸，单次咯血量超过100 mL）。

### 1.4 统计学方法

采用SPSS 21.0进行实验数据处理与分析，经正态性检验和方差齐性检验，呈正态分布的计量资料用Mean±SD表示，进行两个独立样本的t检验；呈非正态分别的计量资料以中位数（范围）[Median (range)]表示。两个独立样本的比较采用Mann-Whitney U检验。计数资料以n（%）表示，组间比较采用χ^2^检验。所有检验均为双向，以P<0.05为差异有统计学意义。

## 2 结果

### 2.1 临床资料汇总

300例患者中共有276例确诊为肺癌，快速HE染色组140例，DQ染色组136例。两组患者的年龄和性别构成无统计学差异（P>0.05）（表1）。但癌种间的性别构成统计学差异明显（男性183例，女性93例，P<0.01）：腺癌182例，男性100例，女性82例；鳞癌56例，男性50例，女性6例；SCLC 38例，男性33例，女性5例。癌种间年龄构成无统计学差异（P>0.05）。

**表 1:** 两种C-ROSE染色诊断效能指标的比较

Items	Rapid HE staining group	DQ staining group	*χ*^2^/z	*P*
*n*	140	136		
Gender (Male/Female)	87/53	79/57	0.473	0.492
Age (median, range, yr)	66 (36-89)	65 (16-91)	-0.521	0.603
NSCLC/SCLC	118/22	120/16	0.906	0.341
The concordance between C-ROSE and tissue pathological diagnosis rate	95.7% (134/140)	97.8% (133/136)	0.946	0.331
NSCLC	95.8% (113/118)	98.3% (118/120)	1.377	0.241
SCLC	95.5% (21/22)	93.8% (15/16)	0.054	0.816
Rapid staining time (median, range, s)	160 (120-200)	120 (100-180)	-5.324	<0.001
Cytology specimen peeling rate	1.4% (2/140)	0% (0/136)	1.957	0.162
Biopsy time (median, range, min)	16 (10-25)	17 (10-26)	-0.411	0.681
The incidence of serious adverse reactions	2.9% (4/140)	2.2% (3/136)	0.118	0.731

C-ROSE: cytological rapid on-site evaluation; NSCLC: non-small cell lung cancer; SCLC: small cell lung cancer.

### 2.2 取材成功率

所有患者均穿刺取材成功，并进行了C-ROSE，均无二次活检（图1，图2）。

**图1:**
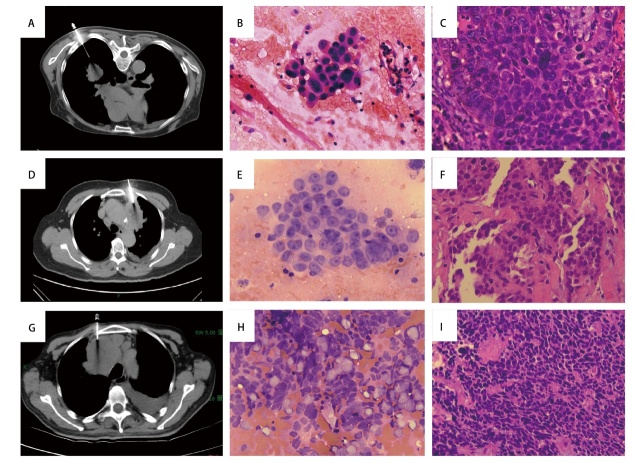
不同类型肺癌组织C-ROSE（快速HE染色）。A：左下肺癌穿刺活检 ；B：快速HE染色，×400：倾向鳞癌；C：组织病理（HE染色，×400）：鳞癌；D：左上肺癌穿刺活检；E：快速HE染色，×400：倾向腺癌；F：组织病理（HE染色，×400）：腺癌；G：左上肺癌穿刺活检；H: 印片快速HE染色，×400：倾向小细胞癌；I：组织病理（HE染色，×400）：小细胞癌。

**图2:**
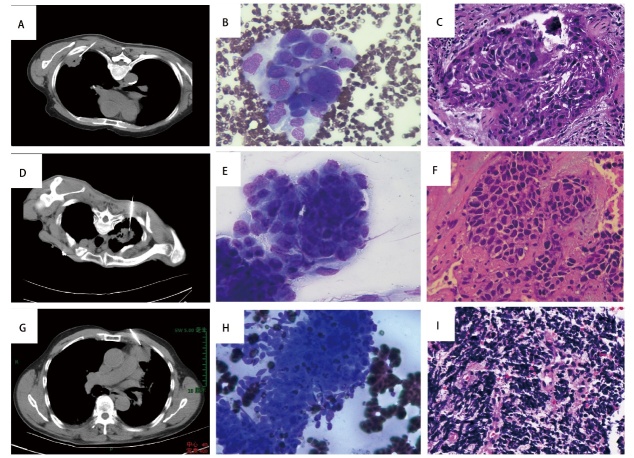
不同类型肺癌组织C-ROSE（DQ染色）。A：左下肺癌穿刺活检；B：DQ染色，×400：倾向鳞癌；C：组织病理（HE染色，×400）：鳞癌；D：右上肺癌穿刺活检；E：DQ染色，×400：倾向腺癌；F：组织病理（HE染色，×400）：腺癌；G：左上肺癌穿刺活检；H：DQ染色，×400：倾向小细胞癌；I：组织病理（HE染色，×400）：小细胞癌。

### 2.3 活检时间

快速HE染色组中位活检时间为16 min，DQ染色组为17 min。两组活检时间无统计学差异（P>0.05）（表1）。

### 2.4 C-ROSE染色时间

快速HE染色组中位染色时间为160 s，DQ染色组为120 s。两组染色时间有统计学差异（P<0.001）（表1）。

### 2.5 染色脱片率

总脱片率为0.7%（2/276），2例均存在组织坏死，两组的脱片率无统计学差异（P>0.05）（表1）。

### 2.6 病理诊断符合率

以最终组织病理作为金标准，评估C-ROSE与组织病理诊断的符合率（图1，图2）。最终组织病理诊断238例NSCLC（腺癌182例，鳞癌56例），SCLC 38例。C-ROSE与组织学诊断符合率为96.7%（267/276），其中NSCLC诊断符合率为97.1%（231/238），SCLC诊断符合率为94.7%（36/38）。两组的C-ROSE与最终组织病理诊断符合率无统计学差异（P>0.05）（表1）。

### 2.7 严重不良反应发生率

60 例患者（21.7%）发生了不同程度的气胸和咯血，其中气胸47例，咯血15例，均为穿刺活检相关并发症。发生严重不良反应有7例（2.5%），6例为胸腔置管引流的气胸和1例大咯血。两组的严重不良反应发生率无统计学差异（P>0.05）（表1）。

## 3 讨论

肺癌的治疗方案须依据病理诊断、分期诊断、驱动基因和免疫检查点等综合情况来制定^[2]^。实施小样本微创取材是诊断肺癌的关键技术之一，尤其对于无手术机会的晚期肺癌患者，获得足量、优质的组织样本量对于患者的诊疗至关重要。CT引导肺穿刺活检是肺癌小样本取材的关键技术之一^[4,7]^。其优势在于：取材成功率高、可重复取材且取材量大、适宜推广性好。目前国内外肺癌指南^[8⇓-10]^均将其作为I类活检方法进行推荐，但在临床实践中仍存在以下问题：（1）有时难以判断穿刺路径和取材部位是否合适，尤其是小病灶或合并肿瘤性坏死；（2）是否合并其他疾病，如炎症、机化、肺不张等；（3）出血和气胸的并发症发生率较高^[11]^，直接影响活检质量，干扰操作。

在诊断性介入肺脏病学操作中，ROSE技术是一项实时伴随活检过程的快速现场细胞学判读技术；在基本不损失标本的前提下，将部分取材进行细胞学涂片，迅速染色，通过显微镜综合临床信息立即判读^[6,12,13]^。ROSE的主要目的包括：评价取材满意度、形成初步诊断或缩小鉴别诊断范围、实时指导介入操作以降低穿刺活检的失败率和避免二次活检等^[14]^。在取材过程中，如通过ROSE判断标本满意，操作即可终止，达到节省操作时间、减轻患者痛苦及降低并发症发生率。因此，有效应用ROSE技术有助于小标本的合理送检和避免不必要的检验和检查，提升效率。ROSE技术目前在呼吸内镜伴随活检中应用较广泛^[13⇓⇓⇓-17]^，包括中央气道内直视活检和透壁针吸活检、外周病变透壁肺活检等；而在CT引导下经皮肺活检术中的应用较少^[18]^。

ROSE快速染色方法有多种^[17⇓-19]^。理想的ROSE染色方法应兼顾速度、制片、染色的方便性以及染色质量，但因缺少相应的随机对照研究而无定论。目前最常用的快速染色同时也是世界卫生组织（World Health Organization, WHO）优先推荐的快速染色方法是DQ染色。但是因大部分临床医师不具备现场细胞学的判读能力，大部分病理科医师因不熟悉DQ染色而更倾向使用HE染色，使得C-ROSE在临床推广受限。本研究针对周围型肺癌穿刺活检标本分别使用快速HE染色^[19,20]^和DQ染色进行C-ROSE，并与最终病理检查结果对比，评估两种快速染色方法的临床应用价值，为C-ROSE技术扩大推广提供一定的依据。

为保证取材质量和控制活检次数，本研究统一采用18 G同轴自动全槽活检枪（美创）。将移动ROSE平台推入CT室，在经皮肺活检术中同步实施ROSE，制片方式采用组织条翻滚涂片法，避免挤压导致标本损失、涂片过厚或厚薄不均。研究结果显示，快速HE染色组的染色时间虽长于DQ染色组，存在显著差异，但单次ROSE时长均低于3 min，且两组的活检总时间、病理诊断符合率无显著差异，提示两种染色方法均可用于快速现场细胞学；从染色速度而言，经典的DQ染色操作步骤简便，因而优于快速HE，对于活检风险较高的患者，使用DQ染色可在控制活检次数的同时，更高效地为操作医生提供现场细胞学信息，节约操作时间。快速HE染色标本中血液成分较多时固定不充分，染色过程中较易出现脱片，操作过程中可以通过扩大涂片面积、减少涂片厚度，来降低或避免脱片。快速HE染色的优势在于：对于较厚的涂片标本，能够更清晰地观察组织细胞结构排列；而DQ染色对于厚涂片标本渗透性较差，且更易褪色或变色，不利于长期保存；快速HE染色使用封片，涂片稳定性和图像质量优于DQ染色；镜下可直接观察到细微结构，在兼顾快速现场细胞学评价的同时，更有利于病理科医师快速出具细胞病理学诊断报告，一举两得。两组取材成功率均为100.0%，所有患者均未因标本取材不充分实施二次活检；两组的严重不良反应发生率无统计学差异，与文献^[21⇓-23]^报道相仿；ROSE技术的介入确实让术者摆脱了介入取材质量盲目性困扰的问题，有效避免二次活检，且安全性良好。

针对周围型肺癌的CT引导肺穿刺活检，伴随进行C-ROSE安全、有效，与组织病理符合率高。在临床工作中，两种染色均可满足C-ROSE需求，可根据实际情况合理选择^[21]^。对于可熟练使用DQ染色的病理医师和临床医师而言，DQ染色仍可作为肺穿过程中的首选C-ROSE方式；对于活检风险高、需要控制活检次数和活检时间者，也建议优先选择DQ染色；而快速HE染色更符合当前国情，对于不熟悉DQ染色的病理科医师和临床医师而言，又需要细胞病理学现场支持者，可优先选择快速HE；有条件可同时配备两种染色液，术中灵活选择，病理医生可同时完成C-ROSE和细胞病理学诊断，提升诊断效率。

**Competing interests**

The authors declare that they have no competing interests.
